# Relative effects of seed mix design, consumer pressure, and edge proximity on community structure in restored prairies

**DOI:** 10.1002/eap.3083

**Published:** 2025-01-19

**Authors:** Riley B. Pizza, Nash E. Turley, Lars A. Brudvig

**Affiliations:** ^1^ Department of Plant Biology Michigan State University East Lansing Michigan USA; ^2^ Ecology, Evolution, and Behavior Program Michigan State University East Lansing Michigan USA; ^3^ Department of Entomology Pennsylvania State University State College Pennsylvania USA

**Keywords:** admixture provenancing, consumer pressure, context‐dependency, edge effects, granivory, herbivory, restoration, seed mix design

## Abstract

A central goal of ecosystem restoration is to promote diverse, native‐dominated plant communities. However, restoration outcomes can be highly variable. One cause of this variation may be the decisions made during the seed mix design process, such as choosing the number of species to include (sown diversity) or the number of locations each species should be sourced from (source diversity, manipulated to affect genetic diversity). The effects that seed mixes have on plant communities may be further modified by other factors at the restoration site, including edge proximity and consumer pressure. Few studies have evaluated both these seed mix attributes together, and none have done so while accounting for realistic restoration site attributes. To address this research need, we conducted a prairie restoration experiment where two aspects of seed mix design (sown diversity and source diversity) and two restoration site factors (edge proximity and vertebrate granivore/herbivore consumer access) were manipulated across 12 replicate fields. We found that when seed mix design impacted plant community structure, these effects were dependent on consumer access or edge proximity and were more prominent after one versus five growing seasons. Low seed source diversity plots had more sown species than high source diversity ones, but only when consumers had access. Similarly, low species diversity plots had higher richness and cover of species included in both the low and high species diversity mixes, but this effect weakened over time. Additionally, plots with high species diversity were buffered from the typically detrimental effects of edges and consumers, although this did not always result in greater sown species abundance. Unexpectedly, plots with the most sown species were those sown with either low source diversity or low species diversity seed mixes, perhaps due to lower seeding rates of reliably establishing species. Our results illustrate how the influences of seed mix design on restored plant communities can be highly contingent on factors like edges, consumers, and time.

## INTRODUCTION

Ecological restoration is a key tool to reverse widespread habitat loss (UNEP, 2019) and increase global biodiversity (Benayas et al., 2009). Commonly, a first step in these projects is to reestablish a native‐dominated plant community, often by adding seeds to ensure that target species arrive (Kimball et al., 2015). When developing a seed mix to use in a restoration effort, managers must decide how many species to include (Barak et al., 2021), and where each species is sourced from (Bucharova et al., 2019; Meissen et al., 2020). Yet, how these two axes of seed mix design together influence plant community structure remains largely unknown, and their effects may depend on conditions at restoration sites.

Manipulating both aspects of seed mix design together at a restoration site could influence plant communities in additive or interactive ways. Increasing the number of species in a seed mix can increase the number of target species that establish (Barr et al., 2017; Larson et al., 2011; Minor et al., 2021) and the diversity of the restored plant community (Kaul & Wilsey, 2021). Similar effects could be seen by increasing the number of places each species is sourced from to increase genetic diversity. For example, if species from different sources differ phenotypically, and each species can occupy a wider niche width (Roughgarden, 1972), species that would otherwise competitively exclude one another can instead coexist (Fridley et al., 2007; Vellend & Geber, 2005; Whittaker, 1975). Higher source diversity seed mixes can also increase the probability that at least some of the seeds will be adapted to restoration site conditions (Kettenring et al., 2014). The number of species and sources in a seed mix could also interactively affect plant communities. For example, adding additional sources may broaden the niche width of an already dominant species, making it even more dominant (Vellend & Geber, 2005). Thus, additional species included in a seed mix may initially establish, but ultimately be outcompeted by other over‐dominant species (Grman et al., 2021). However, few studies have simultaneously manipulated species and source diversity in plant communities (but see Fridley et al., 2007), and none have done this in a realistic restoration context, so it is unclear whether these metrics of seed mix design will interact, nor how this could affect the plant community at a restoration site.

Additionally, the effects seed mix design has on community structure may be contingent on factors at the restoration site. These context‐dependencies can result in restoration efforts performed with identical methods producing very different outcomes (e.g., Norland et al., 2015). Thus, understanding how restoration methods are influenced by context‐dependencies can inform restoration practitioners on the efficacy of certain methods under the realistic restoration conditions. While there are many abiotic and biotic factors that can influence restoration outcomes (Brudvig, 2017), two important factors during seed‐based restoration in this system are edge effects and consumer pressure (granivory and/or herbivory). At a restoration site, edges are primary areas of invasion of non‐sown species (Vila & Ibanez, 2011) and high levels of invasion can reduce sown species abundance, especially in prairie systems (Warren et al., 2002). If increasing either species or source diversity in a seed mix expands the niche space that sown species occupy, this can reduce invasion at the edges of sites (Kennedy et al., 2002) subsequently increasing restoration success. Consumer pressure can also influence plant species abundances and persistence during restoration (Rebollo et al., 2013): high herbivore pressure has been shown to decrease plant species richness by as much as 85% (Xu et al., 2023), and granivory has been shown to significantly decrease plant establishment in prairie restorations (Pellish et al., 2018). The effects that these consumers have on restored plant communities may be modified by seed mix design. For example, if there are phenotypic differences in seed morphology resulting in only some seed sources being desirable by consumers (Howe & Brown, 1999), high source diversity plantings may be buffered from the detrimental impacts of consumers. Alternatively, if herbivory is higher when more species or sources are sown in a seed mix (Drescher & Nolan, 2023), high diversity seed mixes may have low sown species establishment at restoration sites with strong consumer pressure. However, few studies have empirically tested how seed mix design effects are mitigated by these site‐specific factors at the restoration site (Barr et al., 2017; Cook‐Patton et al., 2011).

Finally, the ways seed mix decisions impact plant communities may vary temporally. For example, increasing the number of species in a seed mix may initially result in a community with greater sown diversity, but these effects may fade with time as species diversity declines due to competitive exclusion (Barber et al., 2017, 2019). Source diversity may show the opposite pattern: processes such as niche partitioning may take time to play out (Kettenring et al., 2014). Thus, restorations with low or high source diversity may appear similar at the onset of restoration, but high source diversity plantings may end up with greater sown diversity. Moreover, since previous research has demonstrated that both edge proximity (Debinski & Holt, 2000; Porensky et al., 2012) and consumer effects (Barber et al., 2017) vary temporally, the ways in which these factors modify the effects of seed mix diversity are unlikely to be consistent over time. However, previous research on the effects of seed mix design has typically surveyed communities at only one timepoint, and studies that surveyed communities over time (Galliart et al., 2019; Larson et al., 2011, 2013) did not account for context‐dependencies. Understanding the long‐term effects of manipulating species diversity and source diversity in a seed mix, while also considering how the effects of these decisions can be modified by external factors, will help us better predict how seed mix designs can influence restoration outcomes.

To test how manipulating two aspects of seed mix design, species and source diversity, can influence plant communities undergoing restoration, we asked (1) how does increasing seed mix species diversity and source diversity, both separately and interactively, affect the number of species and their abundances in a plant community. Further, to understand the interactions between seed mix design decisions and context‐dependencies, we asked (2) how are the effects of these seed mix aspects contingent on edge proximity, consumer pressure, and temporal dynamics. To address these questions, we established a prairie restoration experiment that factorially manipulated the number of species and seed sources in seed mixes sown across 12 fields. We coupled this large‐scale experiment with experimental vertebrate exclusion at both the center and edge of each site. We surveyed plant communities twice: once during the first growing season to examine how decisions made during the seed mix design process can impact initial plant establishment, and again 5 years later to see whether these effects persisted over time.

## METHODS

### Experimental design

**FIGURE 1:**
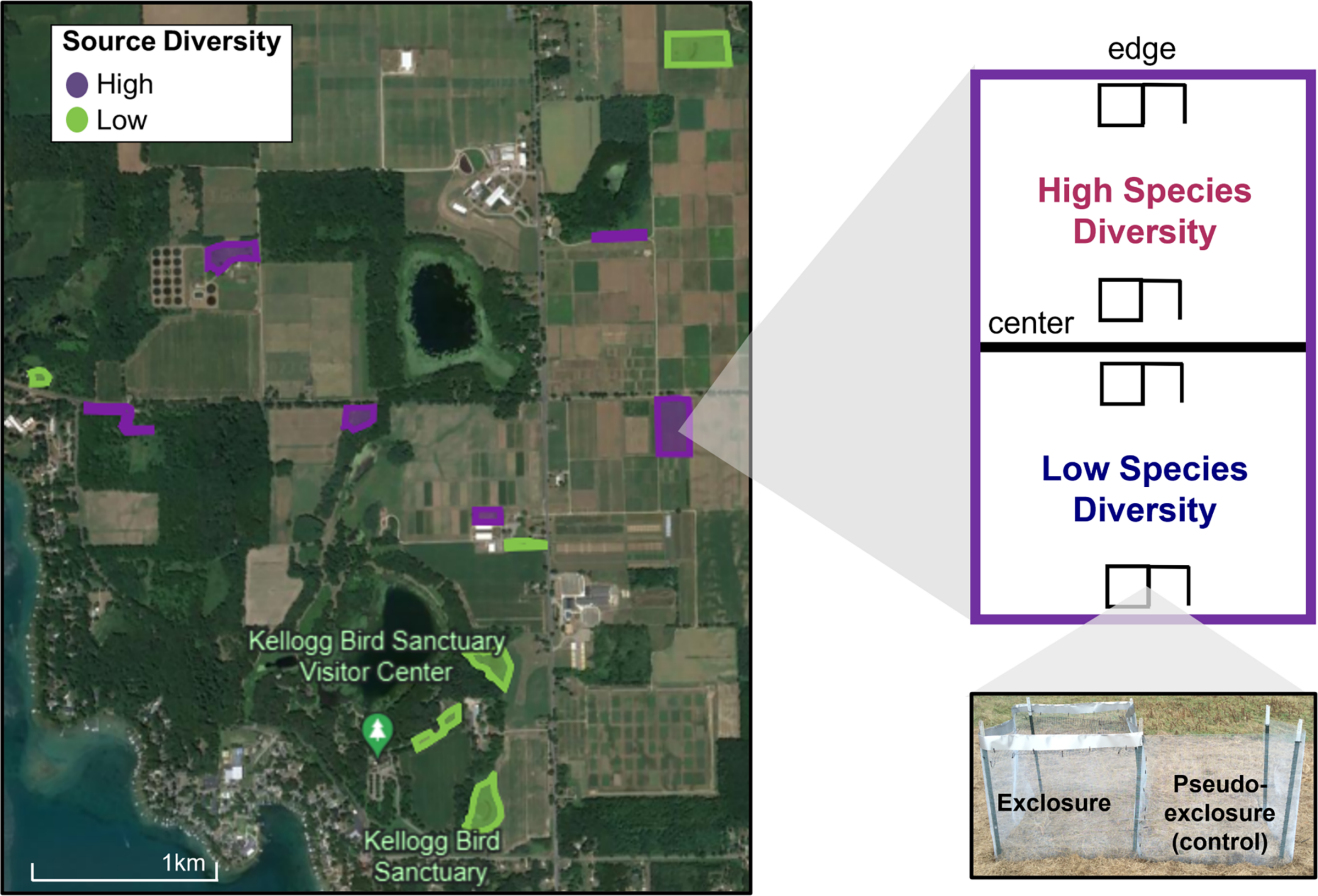
Satellite photograph illustrating the 12 tallgrass prairies undergoing restoration in this study. Fields were seeded with either one or three sources for 12 focal species. Half of each field was seeded with only the 12 focal species (low species diversity) and the other half with the 12 focal species plus 57 more (high species diversity). At the center and edge of each half‐field, 1.5 m × 1.5 m exclosures were built to exclude vertebrate consumers, and each exclosure was paired with a pseudo‐exclosure which was enclosed on only three walls. Plots were surveyed within each of these exclosures and pseudo‐exclosures. The photograph was created using Google Earth, and the image of the exclosures was taken by Nash Turley.

Our experiment used a split–plot design, manipulating seed source diversity at the field level and species diversity at the half‐field level. During the 2015 growing season, we selected 12 fields (ranging from 0.2 to 3.5 ha) around Kellogg Biological Station in southwest Michigan, USA (42.4059, −85.4022) to undergo prairie restoration (Figure 1). All fields were previously cultivated the soil tilled and were old fields dominated by non‐native species before planting. We prepared the fields by mowing the existing vegetation and applying glyphosate herbicide twice before planting, which was successful at killing aboveground plant structures. To initiate restoration, we drilled prairie seeds (~330 seeds/m^2^; see Appendix S1: Table S1 for individual species contributions) into each field following the 2015 growing season with a modified Truax seed drill pulled behind a tractor. We tested the impacts of source diversity with 12 focal species, each of which was purchased from three different locations in the central USA: locally (sourced from the geographically nearest location possible), from more distant, but climatically similar locations (Wisconsin, Minnesota, Illinois, or Iowa), or from southern locations (Missouri or Iowa; Appendix S1: Table S1). We verified with the seed producers that they sourced their seed locally. We randomly assigned sites to be planted either with seed from only one of those sources (low source diversity; *n* = 6; 2 fields of each source), or a seed mix that combined all three sources (*n* = 6). Source diversity was manipulated at the field level to reduce gene flow between high and low source diversity treatments. To test the effects of species diversity, we manipulated the number of species that were added to each half of the field (*n* = 12 in each treatment). We randomly assigned one half of the field to be seeded with only the 12 focal species (low species diversity). To the other, we added those 12 focal species plus an additional 58 species (high species diversity; Appendix S1: Table S1; Figure 1). The additional species, due to limitations on where each species could be purchased from, were sourced from various locations across the Midwest, with each species sourced from a single location. Since the source location of most species differed, we did not consider source identity as a factor in our analyses. All seed mixes contained the same total density of seeds (in grams per square meter).

Two weeks after planting, we assembled 2.25‐m^2^ vertebrate consumer exclosures at the center (at the center point of the half‐field) and edge (1.5 m away from the edge) of each half‐field (*n* = 48; Figure 1). Due to differences in field size, the distance between center and edge plots differed across fields. The plant communities surrounding the fields differed (either a forest edge, a crop field, or a grass lawn) and our sown species were rare or absent from all adjoining habitats. Exclosures consisted of 110–120‐cm‐tall walls of 6.35‐mm wire mesh, buried 10–20 cm underground in an attempt to impede entrance by digging mammals. They also had 10 cm of metal flashing on the tops of the walls to prevent small mammals from climbing in. Each exclosure had a paired pseudo‐exclosure (hereafter referred to as “control”) with only three walls with holes in the bottom to allow entry for vertebrates (Figure 1). We randomized which plot had the exclosure and which one had the pseudo‐exclosure for each pairing. We placed bird netting on top of the exclosures after planting and removed it after the first growing season. While we did not conduct consumer surveys, previous work has shown that granivores (meadow voles, various mouse species, and arthropods) and herbivores (white tailed deer, meadow voles, rabbits, and woodchucks) are important in this system (Anderson et al., 2001; Howe et al., 2006; Linabury et al., 2019).

### Plant community surveys

We surveyed plant communities in September 2016 and July 2021 by placing a 1 × 1 m quadrat in the center of each exclosure and control exclosure. We recorded the identity and visually estimated percent cover of each species. In 2021, to account for variation caused by site‐specific environmental differences, we pooled 10 cm deep soil cores from nine points just outside each exclosure and control exclosure and processed them for soil water holding capacity (the proportionate difference between saturated wet and oven dry weights following Brudvig & Damschen, 2011). We chose this method to characterize the soils as it has been shown to correlate with the abundance of prairie species in our region (Grman et al., 2021; Zirbel & Brudvig, 2020) and with other soil attributes such as soil organic matter and Nitrogen content (Bordoloi et al., 2019).

### Data analysis

We performed all analyses in R studio using R version 4.2.3 (R Core Team, 2024). We used the inverse Simpson's diversity to measure species diversity (Clarke et al., 2014) and its corresponding evenness metric (Smith & Bastow Wilson, 1996) since these metrics are commonly used when species dominance is predicted to be important in community assembly (Morris et al., 2014). Since we expected communities to shift tremendously due to succession in between the first and fifth growing season regardless of our measured factors, we did not include year as a fixed effect in our models. Instead, we ran each model twice, once for each year data was collected.

### Univariate analyses

We analyzed species richness, evenness, Simpson's diversity, sown species richness, and focal species richness (species that were manipulated in the seed sourcing treatment) using separate mixed effects linear models for each response variable (Bates et al., 2014). All models included the following predictors: seed source diversity, species diversity, edge proximity, consumer access, as well as every two‐way interaction between these factors. We also included soil water holding capacity as a covariate. To account for the split–split–plot design, we included a random factor that nested exclosure status, edge proximity, and seed mix diversity. After running the analyses, we removed the interaction between edge proximity and exclosure status, as this interaction was never significant in any analyses, nor did it directly relate to our research questions. All corresponding statistical results are reported in Appendix S1: Table S2.

To establish if seed mix design influenced dominant species, we also analyzed the cover data for the most common species in our plots. We defined a species as “common” if it was found in at least half (45) of our plots in a given year. In 2016, common species were *Daucus carota*, *Elymus repens*, *Plantago lanceolata*, and *Solidago canadensis*. In 2021, common species were two weedy non‐native species, *El. repens* and *Poa pratensis*, and one weedy native species *S. canadensis*. Although these species were not locally abundant in every plot, they often were: the cover of these species in each plot often exceeded 40%, and cover reached up to 95% in some plots. Since no sown species were ever common by our definition, we also analyzed the most abundant sown species 5 years after establishment (when they were most prevalent): *Andropogon gerardii* (found in 24 plots) and *Echinacea purpurea* (31 plots). All species‐level models were over dispersed but not zero‐inflated (all zero‐inflation tests *p* > 0.6; DHARMa package version 0.4.6; Hartig & Hartig, 2017), so we used a negative binomial mixed model (Brooks et al., 2017). Due to limitations of this model, the coefficients are not standardized. All corresponding statistical results are reported in Appendix S1: Table S3.

### Multivariate analyses

To test the effects of seed mix design and other factors on community composition, we conducted a permutational multivariate analsis of variance (PERMANOVA) using the “adonis2” function (Vegan package version 2.6‐4; Okansen, 2010). Because of our split–split–plot design (the source diversity treatment being applied to the entire field) and limitations of the “adonis2” function, we could not analyze the effects of species diversity, source diversity, edge proximity, and consumer pressure in one model. Thus, we split this analysis into two components: one to test the effects of source diversity and species diversity on community composition, and another to test the effects of species diversity, edge proximity, and consumer access on species covers. We ran the first model using only source diversity, species diversity, and their interaction as fixed effects. We also included field ID as a fixed effect nested within half‐field ID to account for field differences and interpreted it as a random factor, to account for limitations with fitting random effects in the adonis2 function. If any factors were significant in analyses, we ran tests of dispersion on any significant terms using the “betadisper” function (Vegan package version 2.6‐4; Okansen, 2010). We ran the second model with the same factors as all the univariate models, except field ID nested within half‐field ID was used as a fixed effect to correct for the degrees of freedom not accounted for without using a nested random effect (owing to limitations with fitting random effects in the adonis2 function). In this model, all permutations were conducted within fields. Both models had 10,000 permutations. To visualize differences in plant communities based on the interaction between source diversity and species diversity, we used a canonical analysis of principal coordinates (Anderson & Willis, 2003) of Bray–Curtis dissimilarity matrices using the function “capscale” (Vegan package version 2.6‐4; Okansen, 2010). All corresponding statistical results are reported in Appendix S1: Table S4.

## RESULTS

### Relative importance of factors

**FIGURE 2:**
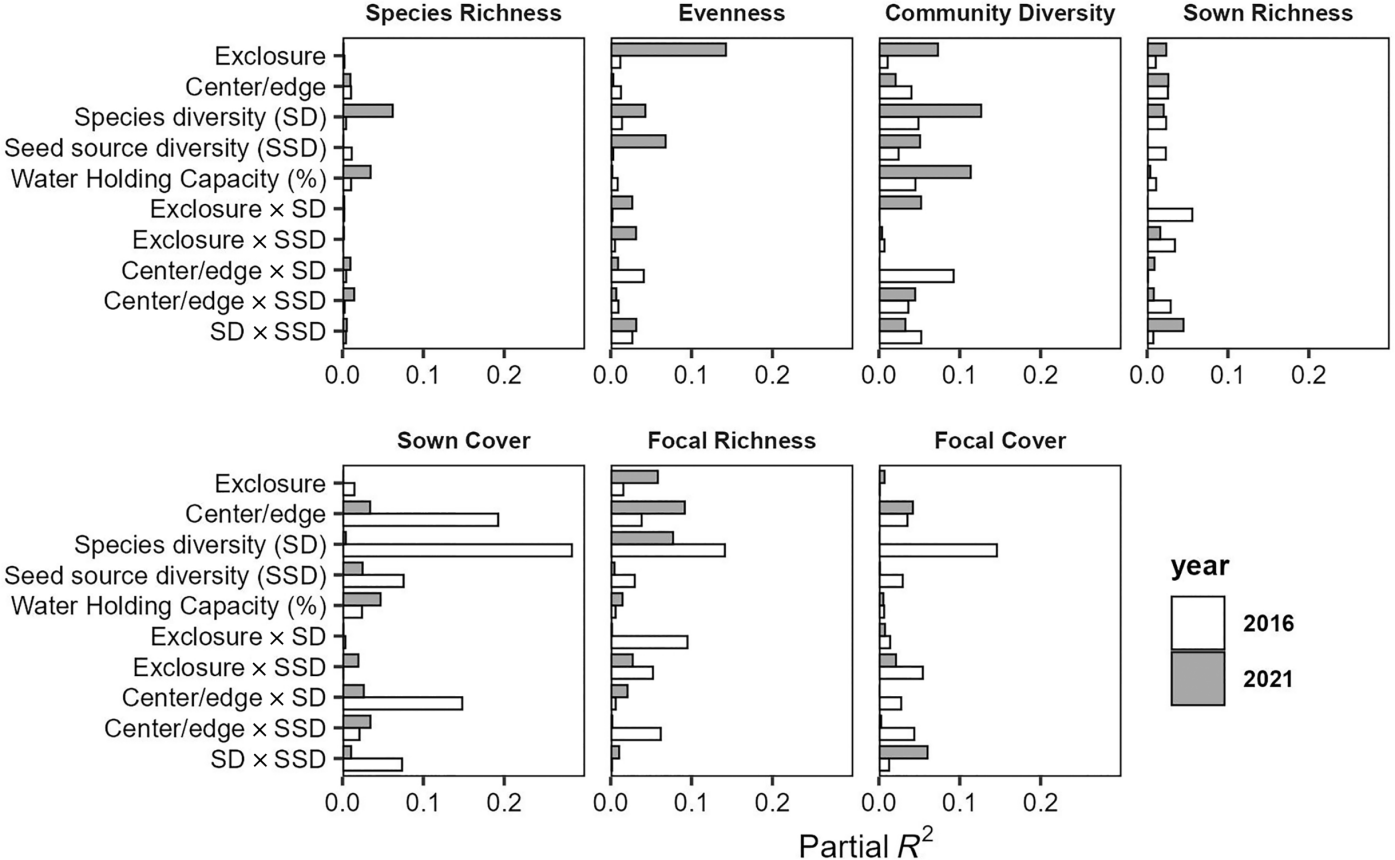
Partial *R* ^2^ values for seven community response variables measured in 2016 and 2021 to quantify the effects of seed mix design, edge effects, consumer pressure, and their interactions on the presence and abundance of species sown into 12 tallgrass prairie fields undergoing restoration. “Sown” refers to any species that were included in either the low or high species diversity seed mix, and “Focal” refers to the 12 species included in both seed mixes where seed source diversity was manipulated.

Overall, our measured factors (source diversity, species diversity, edge proximity, and consumer pressure) had the greatest impact during the first year of establishment, with 83% of models having at least one significant factor, compared with only 43% of models conducted on 2021 data (Appendix S1: Tables S2–S4, Figures S1–S3). Additionally, the amount of variance explained for factors in our models was considerably higher in 2016, especially those related to the species sown into the experiment (Figure 2). Across both years, seed source diversity was a significant factor in 28% of all univariate analyses, but only through interactions with other factors, especially the exclusion of consumers (Appendix S1: Figures S1–S3, Table S2). Seeded species diversity was a significant factor in 58% of analyses, both as a main effect and as interaction terms with consumer exclusion or edge proximity (Appendix S1: Figures S1–S3, Table S2). Edge proximity was a significant predictor in 31% and consumer pressure in 27% of analyses (Appendix S1: Figures S1–S3, Tables S2 and S3).

### Main effects of seed mix design

**FIGURE 3:**
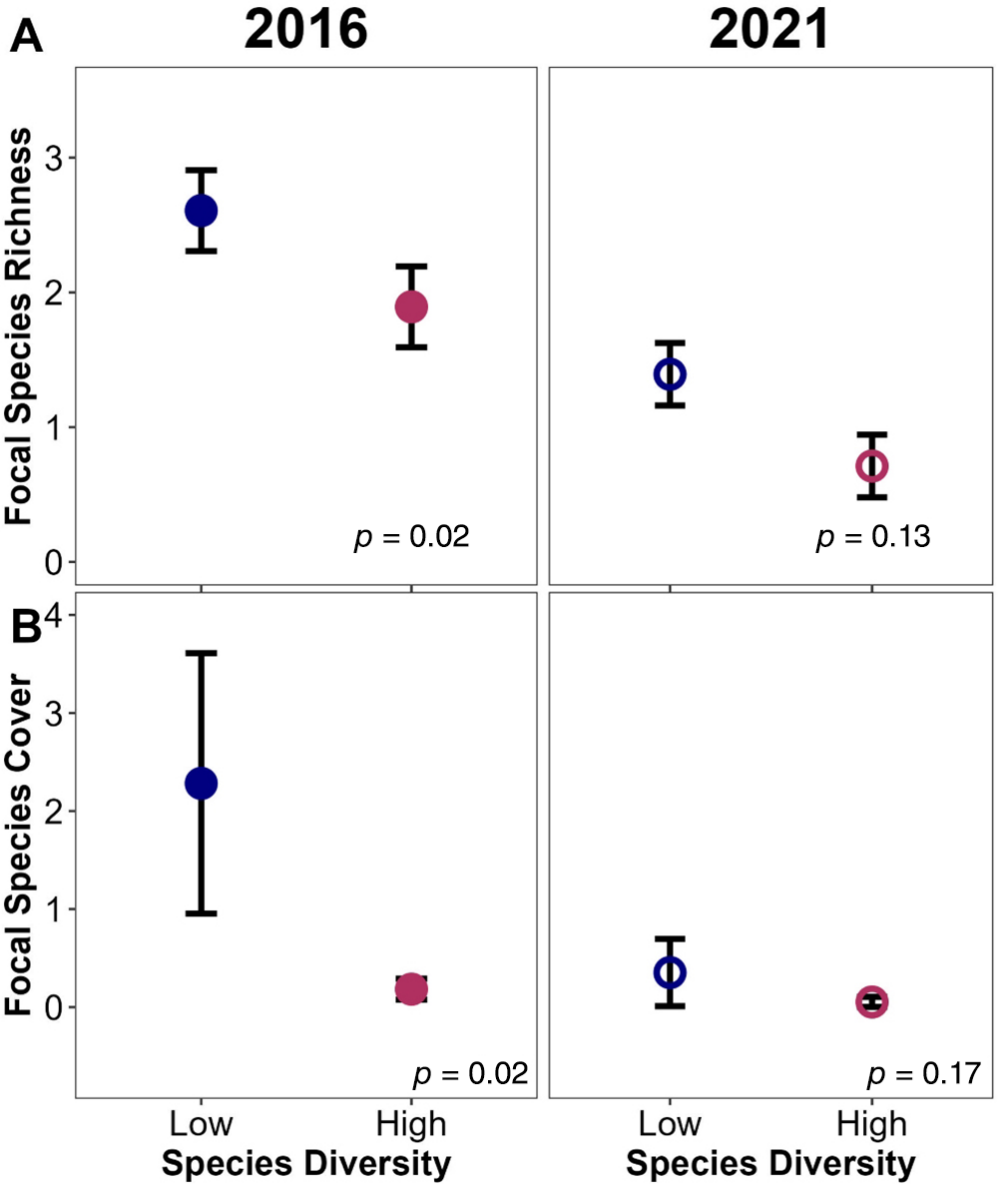
Conditional effects, accounting for other model factors, of the number of species used in a seed mix on cover of (A) focal species richness and (B) focal species cover both in the first growing season (closed circles) and 5 years after establishment (open circles; *n* = 96) in 12 tallgrass prairies undergoing restoration. “Focal” refers to the 12 species included in both the high‐ and low‐diversity seed mixes where seed source diversity was manipulated. Circles indicate mean values calculated with emmeans() and error bars show SE. The *p* values on each panel reflect the species diversity factor significance in each model.

Seed source diversity was never a significant main effect in our community level models (Appendix S1: Table S2). Sown species diversity was a significant main effect, but only for sown species responses. Focal species richness (sown species where seed source was manipulated) was 38% higher in low sown species diversity plots during the first year of growth (Figure 3A). Five years later, there was marginal evidence that there were more focal species in low species diversity plots than in high species diversity plots, although focal species richness was lower than it was during establishment (Figure 3A). There was a more pronounced difference in focal species cover: it was 92% higher (Figure 3B) in low species diversity plots than in high species diversity plots. Five years later, though, this trend disappeared. The interaction term between source diversity and species diversity was never significant in any of our community measures (Appendix S1: Table S2).

### Interactions between seed mix design, edge proximity, and consumer pressure

**FIGURE 4:**
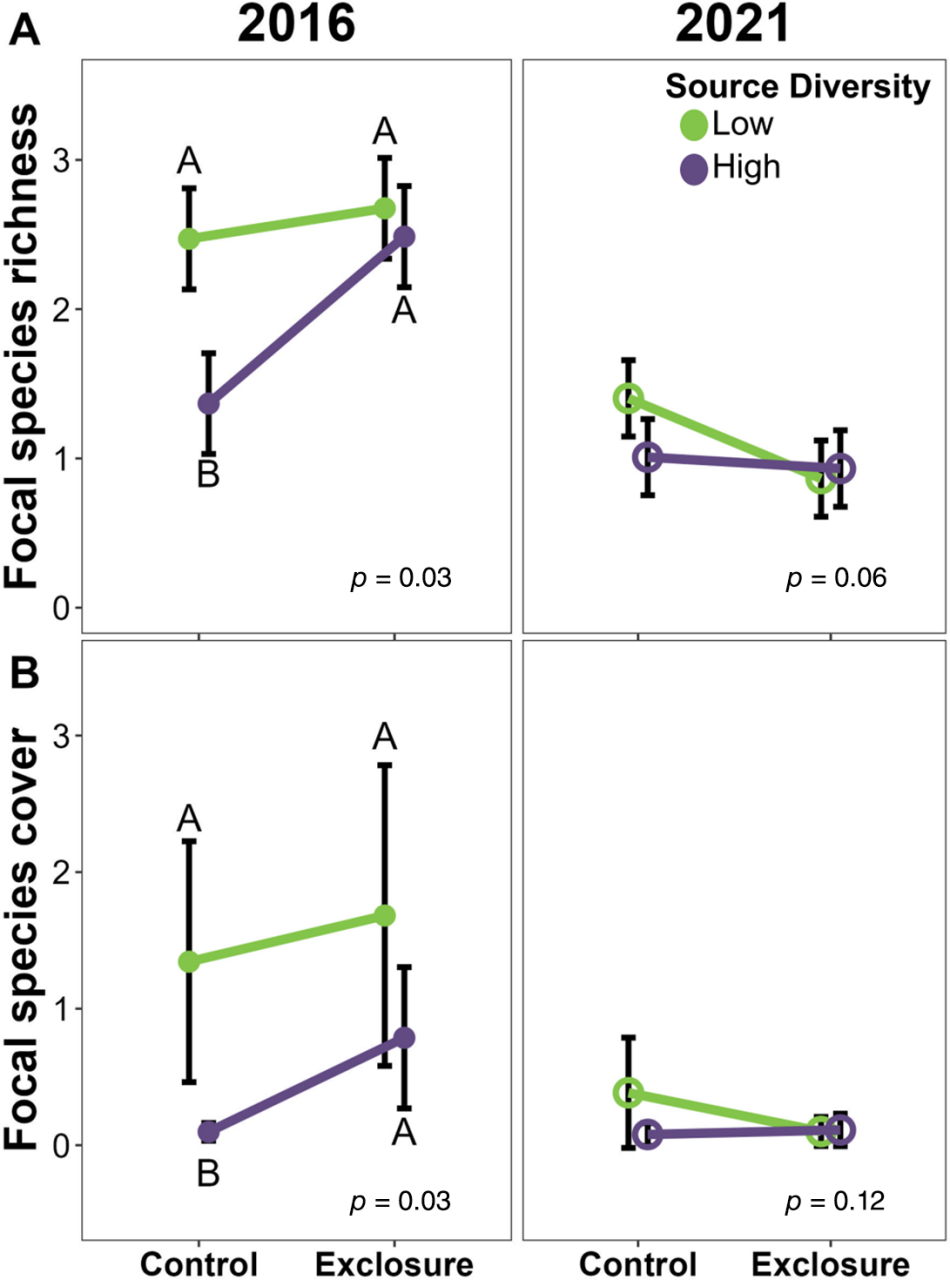
Conditional effects, accounting for other model factors, of the interaction between the number of sources used in a seed mix and whether a plot was excluded from consumers on (A) the number of focal species in a plot and (B) the cover of those focal species both in the first growing season (closed circles) and 5 years after establishment (open circles; *n* = 96) in 12 tallgrass prairie fields undergoing restoration. “Focal” refers to the 12 species included in both the high‐ and low‐diversity seed mixes where seed source diversity was manipulated. Circles indicate mean values calculated with emmeans(), error bars show SE, letters indicate differences between groups based on post hoc tests, and *p* values on plots are for the interaction effect. Plots with no letters indicate either significant effects of the interaction between source diversity and exclosure with no differences in conditional means, or an insignificant interaction effect (see model effect *p* value on each panel).

**FIGURE 5:**
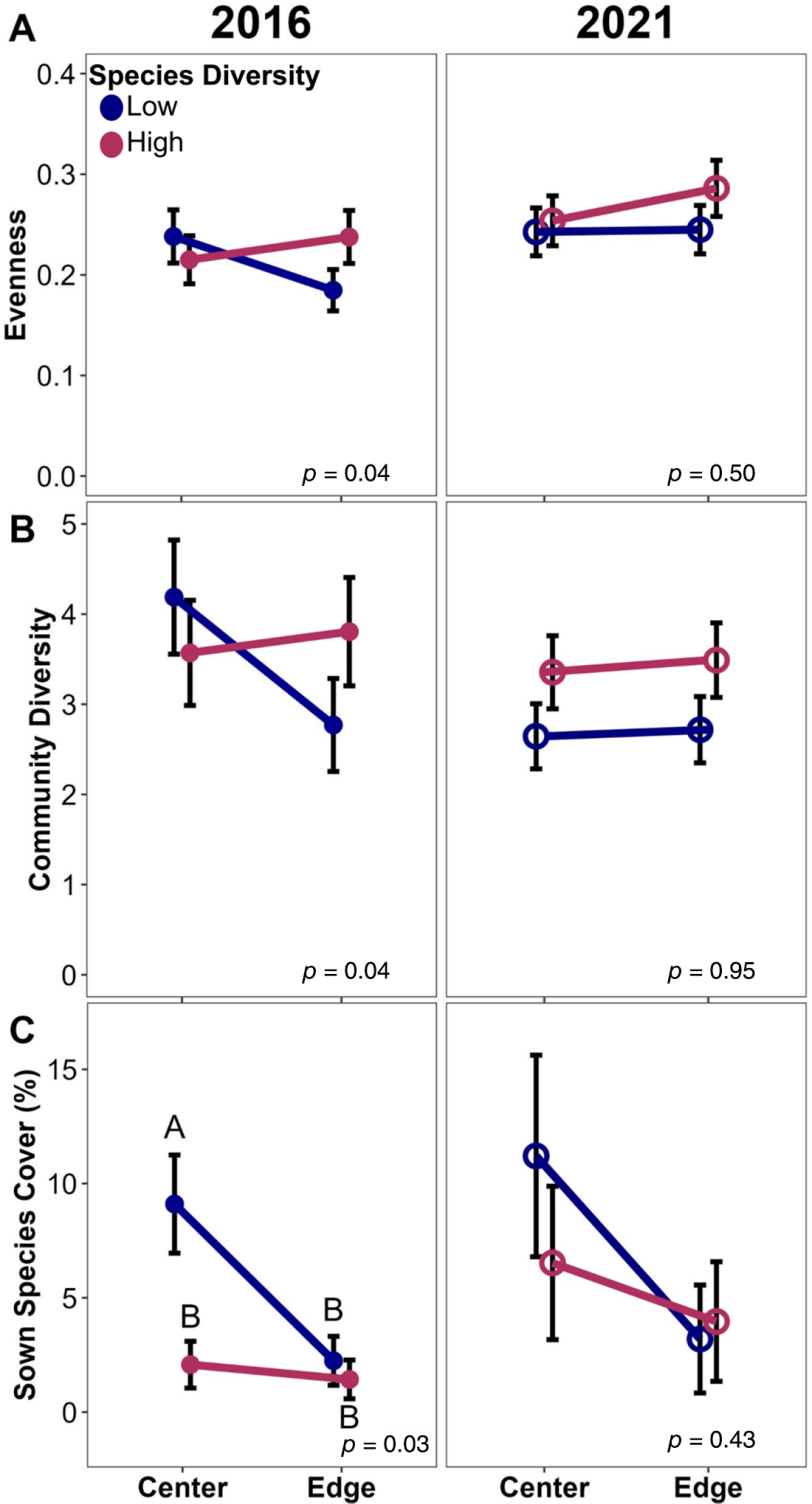
Conditional effects, accounting for other model factors, of the interaction between the number of species used in a seed mix and whether a plot was at the center or edge of a field on the (A) evenness of species, (B) community diversity, and (C) sown species cover both in the first growing season (solid circles) and 5 years after establishment (open circles; *n* = 96) in 12 tallgrass prairies undergoing restoration. Circles indicate mean values calculated with emmeans(), error bars show SE, and letters above bars indicate significance groups. Plots with no letters indicate either significant effects of the interaction between source diversity and exclosure with no differences in conditional means, or an insignificant interaction effect (see model effect *p* value on each panel).

The effects that seed mix design had on plant communities were often dependent on edge proximity and consumer pressure. In the first growing season, low seed source diversity plots had, on average, one additional focal species (sown species where seed source was manipulated), but only in plots that allowed consumer access (Figure 4A). This relationship persisted 5 years later, although the effect was weaker. We observed a similar trend for focal species cover (~1.5% greater cover in low source diversity plots; Figure 4B) and sown species richness (~1 species; Appendix S1: Table S2), but these trends did not persist through 2021. At the center of fields, species evenness was 25% higher (Figure 5A), community diversity 36% higher (Figure 5B), and sown species cover 75% higher (Figure 5C) than at the edge of fields during the first growing season, but only in low species diversity plots. All three of these interactive effects disappeared when plots were surveyed again 5 years later (Figure 5).

**FIGURE 6:**
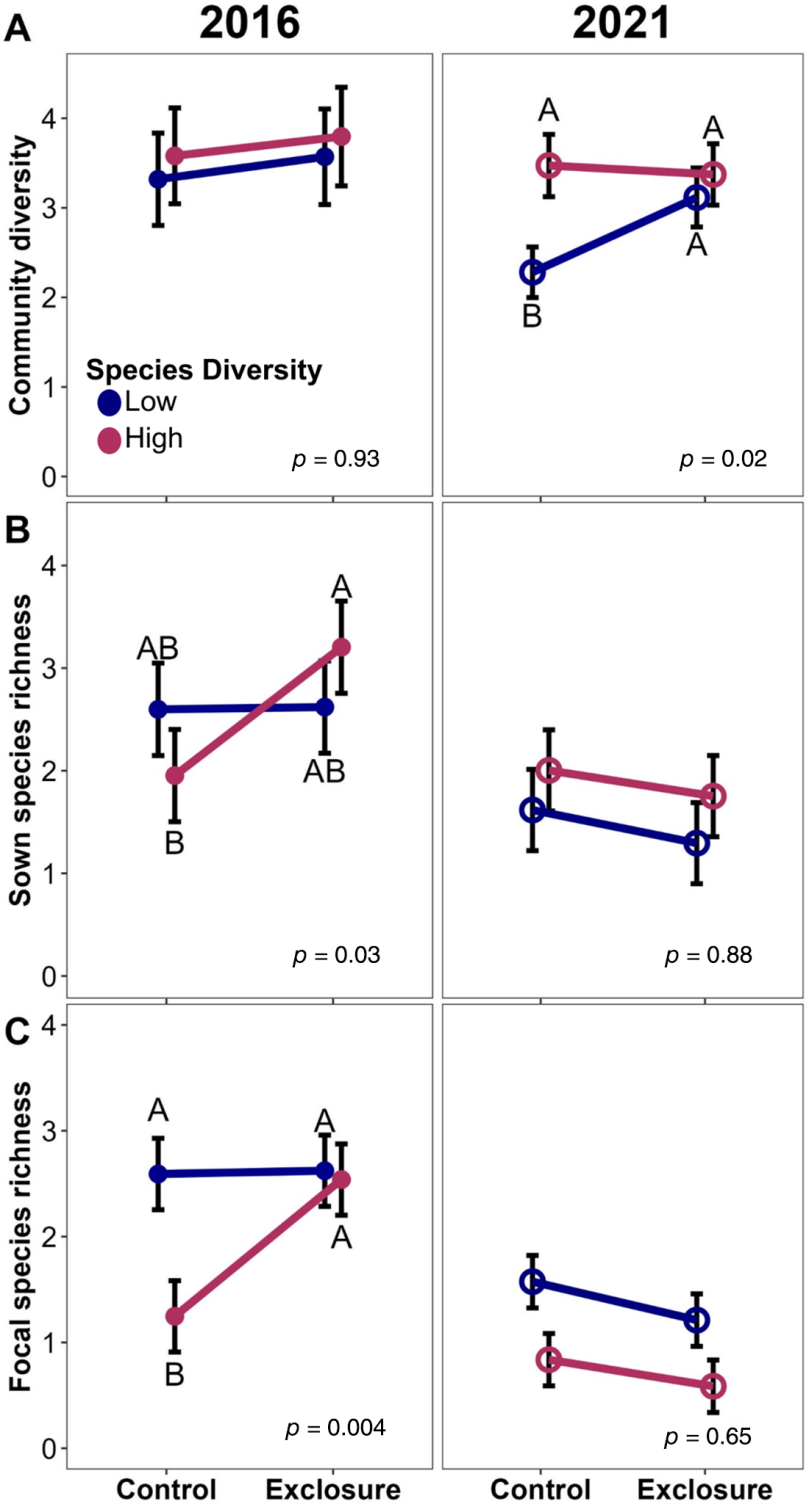
Conditional effects, accounting for other model factors, of the interaction between the number of species used in a seed mix and whether a plot allowed consumer access on (A) community diversity, (B) sown species richness, and (C) focal species richness both in the first growing season (solid circles) and 5 years after establishment (open circles; *n* = 96) in 12 tallgrass prairies undergoing restoration. “Sown” refers to any species that were included in either the low or high species diversity seed mix, and “Focal” refers to the 12 species included in both seed mixes where seed source diversity was manipulated. Circles indicate mean values calculated with emmeans(), error bars show SE, and letters above bars indicate significance groups. Plots with no letters indicate either significant effects of the interaction between source diversity and exclosure with no differences in conditional means, or an insignificant interaction effect (see model effect *p* value on each panel).

There was a more complex relationship between species diversity and consumer pressure: 5 years after establishment, community diversity was 27% higher in plots where consumers were excluded, but only in low species diversity halves (Figure 6A). This trend was not observed during the first growing season. We observed a different trend for sown species: sown species richness was 41% higher (Figure 6B) and focal species richness was 51% higher (Figure 6C) when consumer access was prevented, but now only in high species diversity plots and only during the first growing season.

**FIGURE 7:**
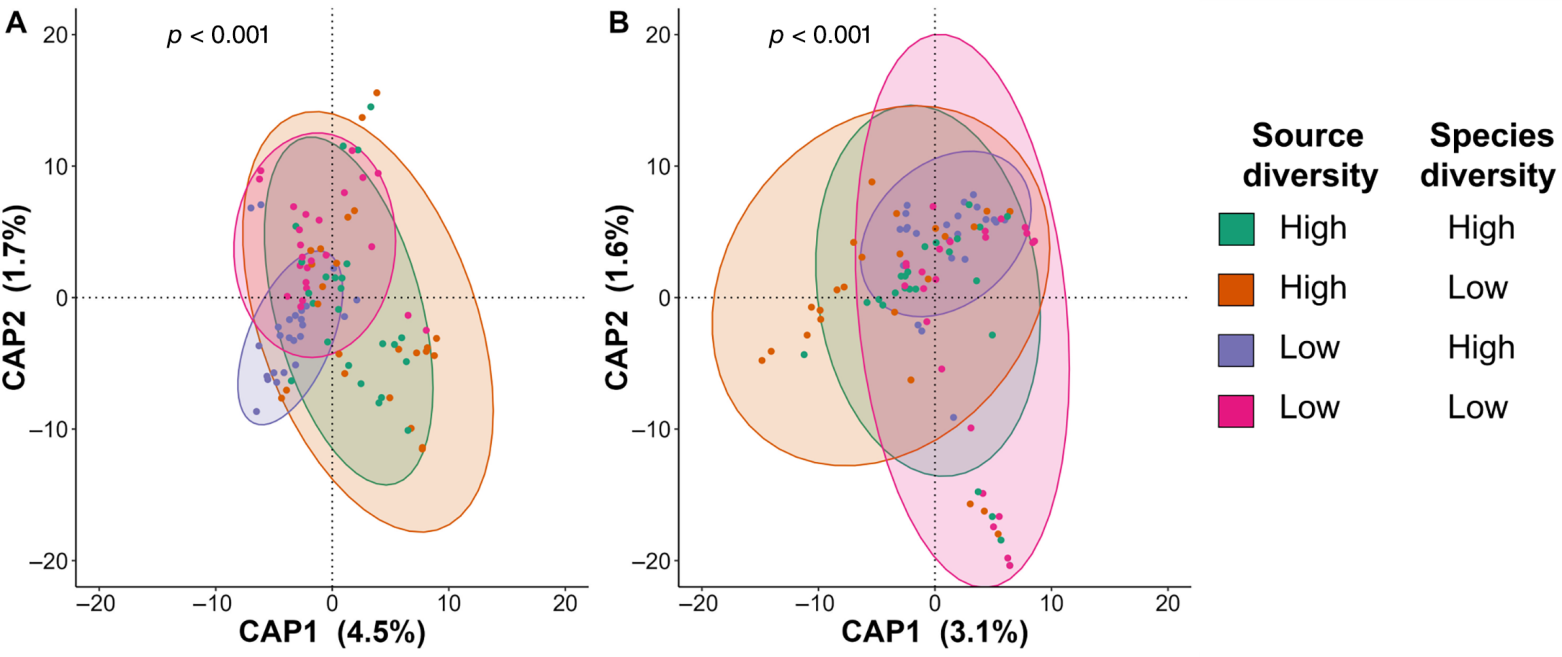
Canonical analysis of principal coordinates (CAP plots) for the interactive effect of source diversity and species diversity in a seed mix on plant community composition during the first growing season (A; PERMANOVA *R* ^2^ = 0.02) and 5 years following establishment (B; PERMANOVA *R* ^2^ = 0.02) in 12 tallgrass prairies undergoing restoration. For full PERMANOVA statistics, see Appendix S1: Table S5.

There was never an interaction between seed source diversity and species diversity for any of our univariate metrics of community structure. However, there was weak evidence that evenness was higher in high seed source diversity fields, but only in low diversity halves during the first growing season (Appendix S1: Table S2). We did, however, observe an interaction between species diversity and source diversity in multivariate analyses of community structure both during initial establishment (Figure 7A) and 5 years later (Figure 7B; Appendix S1: Table S4), although the effect was small (*R* ^2^ = 0.02). There was no difference in multivariate dispersion among treatments in either year (all *p* > 0.15).

### Main effects of context‐dependencies

There were (~1.25) more sown species and marginally greater focal species cover at the center of fields than at the edges during the first year of establishment, and ~1 more species at the center of fields than at the edges 5 years after establishment (Appendix S1: Table S2). Consumer pressure was never significant as a main effect in our models (Appendix S1: Table S2). Finally, water holding capacity was only significant in one analysis, with drier fields having greater community diversity (Appendix S1: Table S2). The field random effect (the 12 locations) explained, on average, 42% (±0.12 SD) of the variation in models.

### Individual species responses

**FIGURE 8:**
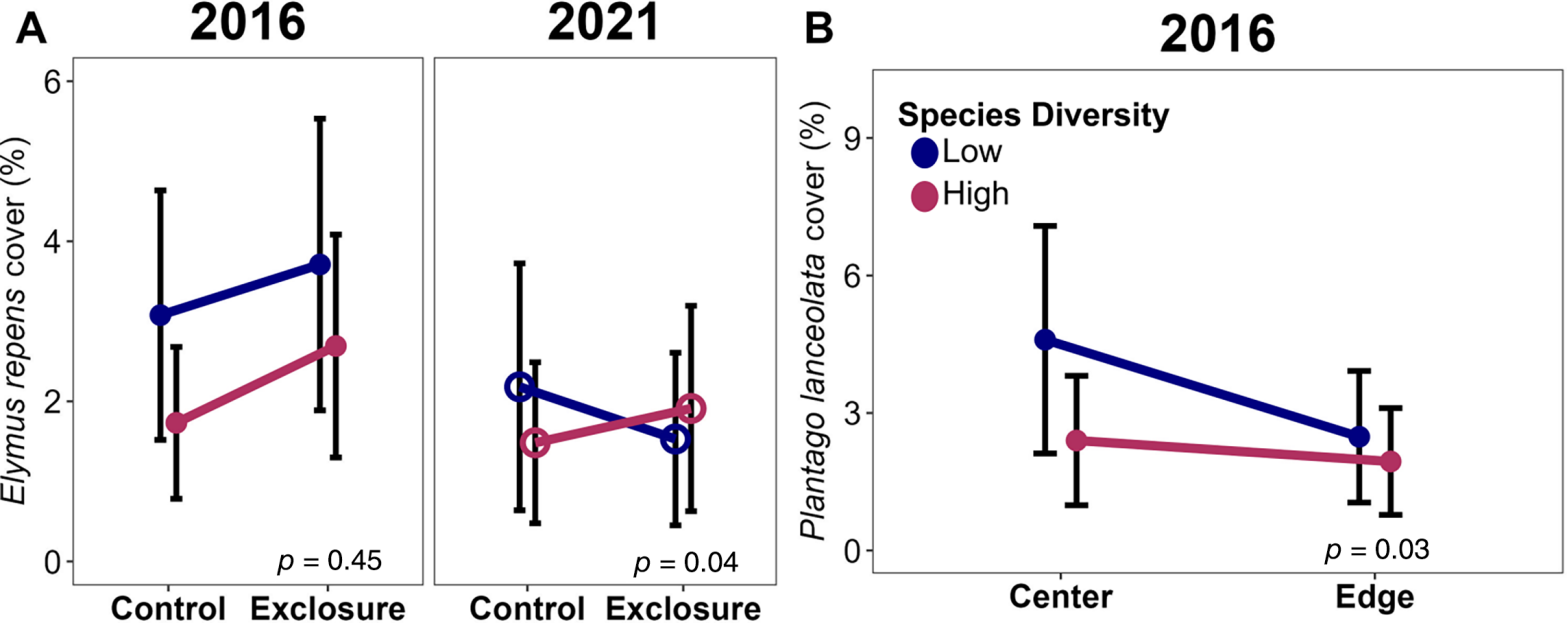
Conditional effects, accounting for other model factors, of the interaction between (A) the number of species used in a seed mix and whether a plot allowed consumer access on the percent cover of *Elymus repens* during the first growing season (closed circles) and 5 years after establishment (open circles) and (B) the number of species used in a seed mix and whether a plot was at the center or edge of a field on the percent cover of *Plantago lanceolata* during the first growing season (open circles; *n* = 96) in 12 tallgrass prairies undergoing restoration. Circles indicate mean values calculated with emmeans(), and error bars show SE.

**FIGURE 9:**
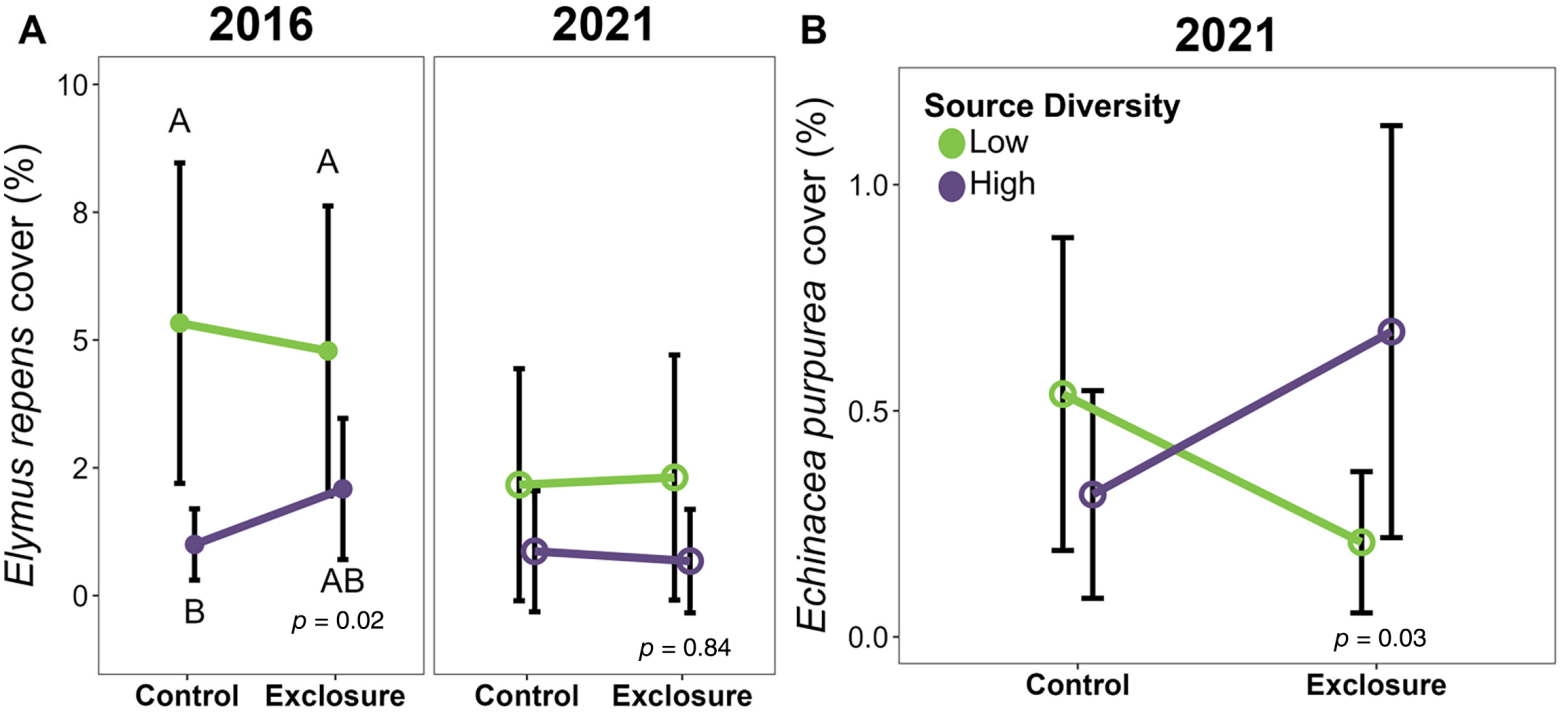
Conditional effects, accounting for other model factors, of the interaction between the number of species and seed sources used in a seed mix and whether plots allowed consumer access on the cover of (A) *Elymus repens* during the first growing season (solid circles) and 5 years after establishment (open circles) and (B) *Echinacea purpurea* 5 years after establishment (*n* = 96) in 12 tallgrass prairies undergoing restoration. Circles indicate estimated mean values calculated with emmeans(), error bars show SE, and letters above bars indicate significance groups. Plots with no letters indicate either significant effects of the interaction between source diversity and exclosure with no differences in conditional means, or an insignificant interaction effect (see model effect *p* value on each panel).

Of the five non‐sown species and two sown species we investigated, three were impacted by our factors: *El. repens*, *P. lanceolata*, and *Ec. purpurea*. The other four species were not impacted by any of our measured factors, although *S. canadensis* was marginally more abundant at the center of sites than at the edges during the first growing season (Appendix S1: Table S3). During the first growing season, the non‐sown species *El. repens* cover tended to be higher in the low source diversity plots, but not significantly. Five years later, though, *El. repens* cover was 30% higher in low source diversity plots when consumers had access, but 22% lower in high source diversity plots (Figure 8A). The cover of *P. lanceolata* during the first growing season was 65% higher at the center of fields than at the edges in low source diversity plots, but edge proximity did not affect cover in high species‐diversity plots (Figure 8B). *El. repens* cover was 30% higher in low source diversity plots than high source diversity plots, but only when consumers were present (Figure 9A) and only during the first growing season. Excluding consumers in plots with high seed source diversity increased *Ec. purpurea* cover by 60%, whereas in plots with low source diversity, it decreased cover by 53% (Figure 9B).

**FIGURE 10:**
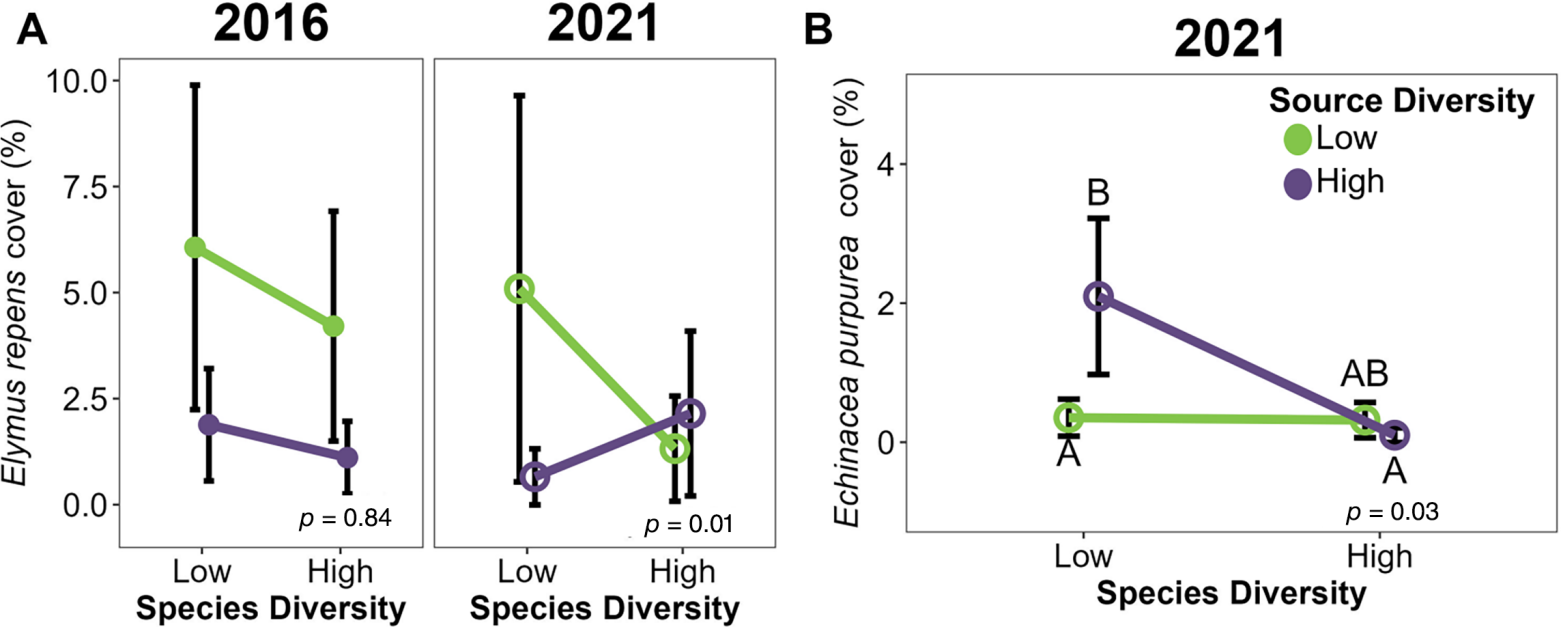
Conditional effects, accounting for other model factors, of the interaction between the number of species and seed sources used in a seed mix on the cover of (A) *Elymus repens* during establishment during the first growing season (solid circles) and 5 years later (open circles) and (B) *Echinacea purpurea* 5 years after establishment (*n* = 96) in 12 tallgrass prairies undergoing restoration. Circles indicate estimated mean values calculated with emmeans(), error bars show SE, and letters above bars indicate significance groups. Plots with no letters indicate either significant effects of the interaction between source diversity and exclosure with no differences in conditional means, or an insignificant interaction effect (see model effect *p* value on each panel).

Two species' covers were impacted by the interaction between source diversity and species diversity, but in different ways. In 2021, in low source diversity fields, there was 31% greater cover of *El. repens* in low species diversity plots than in high species diversity plots, but there is (Figure 10A). Of note, *El. repens* cover was qualitatively higher in the low source diversity plots during the first growing season regardless of sown species diversity. The other species, *Ec. purpurea*, had 87% greater cover in high source diversity plots, but only when species diversity was low (Figure 10B).

## DISCUSSION

To understand the consequences of seed mix design decisions on restored plant communities in a realistic restoration context, we conducted a large‐scale experiment that manipulated both source and species diversity in a seed mix and accounted for site‐specific factors including edge proximity, consumer pressure, and time. Overall, when we found evidence of seed mix design decisions affecting plant community structure, the effects were dependent on consumer access or edge proximity and were most prominent during the first growing season. Specifically, high species diversity plots were buffered from the effects of edges and consumer access, which decreased sown species abundance in low species diversity plots. However, these plots did not consistently have higher sown species richness. Additionally, low source diversity plots tended to have more sown species, especially when consumers had access. Thus, our results suggest that increasing the number of sown species can reduce the effects of site‐specific contingencies, whereas increasing sown source diversity may result in reduced sown species establishment.

### Increasing source or species diversity reduced sown species establishment

Generally, our results did not support our predictions for the ways seed source diversity could influence plant community structure. For example, we expected to see higher focal species richness and cover when plots were seeded with multiple sources, due to increased niche width and reduced interspecific competition (Vellend & Geber, 2005). Instead, focal species richness was lowest in these plots. One of our focal species, *Ec. purpurea* did have greater cover in the high source diversity plots, providing some support that competition could be reduced with higher genetic diversity. Our other prediction was that the number of sown species present in the community could decrease if increased source diversity allowed one of our focal species to become overdominant (Vellend & Geber, 2005). However, since none of our sown species were ever common, our study did not provide a strong test of this hypothesis. Due to the abundance of non‐sown species observed in this study, the sown cover we measured may not have been sufficient to cause some of the hypothesized effects of seed source diversity.

The effects of source diversity were often modified by consumer access. Our 12 focal species were least abundant in high source diversity plantings, but only when consumers had access. This indicates that the effects that source diversity has on restoration outcomes may be due to trophic interactions with other species, rather than inherent properties of the plants themselves. Some possible explanations for this trend are that granivores preferred certain sources over others (Lundgren & Rosentrater, 2007; Perkins et al., 2007), or that high source diversity communities attracted more consumers through either increases in traits desirable to generalist consumers or by increasing the variety of traits to attract a greater diversity of consumers (Castagneyrol et al., 2012; Koricheva & Hayes, 2018; Orrock & Witter, 2010). Interestingly, these trends only persisted during the first growing season: 5 years later, focal species richness and cover were no different between the low and high‐source diversity fields. This suggests that our exclosures had the strongest effects on granivory, which we expect to be important for plant community establishment from seed at the onset of restoration, rather than herbivory, which may become a more important process once the community is established.

Sown species diversity more commonly influenced community structure in our experiment, although it did not always result in more sown species in each plot. In high species diversity plots, we observed lower richness and cover of our sown species, especially the 12 focal species. However, this was contingent on consumer access and edges. In part, this may be a sampling effect: measuring at the plot‐level, we were inherently less likely to come across our focal species that were diluted in the high‐diversity seed mix (Šímová et al., 2013). However, since both plantings had the same seeding density, we would still expect to see equal numbers of sown species. Instead, there is evidence that adding additional species, at the cost of decreasing the amount of seed from reliably establishing ones, ultimately reduced the number of sown species in each plot. For example, *A. gerardii* and *Ec. purpurea*, our most common sown species, were both in the low species diversity mix. Given that adding additional species can be expensive (Schaub et al., 2021), practitioners should carefully consider the benefits of adding additional species if they are unable to do so without reducing the overall seeding rate of the species already in the mix.

### Sown species diversity effects were often contingent on edge proximity and consumer pressure

Although seeding higher diversity seed mixes reduced sown species cover, we also found evidence that these seed mixes can minimize the often‐detrimental effects of edge proximity and consumer pressure. For example, community diversity was highest at the center and lowest at the edges of fields that were sown with fewer species during the first growing season. However, fields sown with higher species diversity seed mixes had consistent levels of community diversity across the center and edges of fields. It is possible that despite high species diversity plots having fewer sown species, the sown species that are present are more functionally diverse, or have traits similar to invading species, allowing for greater niche coverage and less opportunity for non‐sown species to invade and establish (Kennedy et al., 2002). We saw similar patterns when consumer access was manipulated: plots sown with fewer species exhibited high community diversity only when consumers were excluded, but plots sown with more species retained high levels of diversity, even when consumers had access. We expect that, since some species were likely consumed more readily than others (Herrera & Pellmyr, 2009), some species in the low‐diversity mixtures are being lost and not replaced by other seeded species. In the high‐species mixtures, those species are more likely replaced due to functional redundancy in the seed mix (Petchey & Gaston, 2002) maintaining high levels of diversity (Palmer et al., 1997; Tilman & Downing, 1994). Given that edge effects and consumer pressure can lead to unpredictable restoration outcomes, increasing species diversity in a seed mix at sites where these factors are strong, without decreasing the seeding rate of well‐establishing species, could result in more diverse restoration plantings. There are many site‐specific factors that we did not measure that likely influenced the community structure of these restorations (evidenced by the high amount of variation explained by the site random effect in our models). Future studies measuring additional site‐specific context‐dependencies in this experiment, such as the different soil characteristics at each site, may identify additional factors that can influence the impacts of seed mix design on restoration outcomes.

### Manipulating both source and species diversity had species‐specific effects

The interaction between source diversity and species diversity was rarely an important factor in any of our plant community measures. However, it was significant in analyses that accounted for species identity: our multivariate analysis suggested that communities were structured by the combination of the number of sources of our focal species and the number of additional species in the seed mix. Although this interaction did not explain a large amount of variation in community composition, it suggests that species responded differently to the source and species diversity treatment.

There was also some evidence for an interaction between source diversity and seeded species diversity when considering individual species, but only 5 years after planting. *El. repens* cover was lowest when either source or species diversity was increased, suggesting that increasing diversity in the seed mix in any way makes it more difficult for this exotic, invasive species to establish. Since *El. repens* is a long‐lived perennial grass, it took time to become established in the fields, explaining why its cover was not significantly affected by our seed mix in the first year. Future research designed to understand why *El. repens*, unlike any of the other dominant non‐sown species found in our plots, was impacted by seed mix design may illuminate other ways seed mix design decisions can influence restoration outcomes.

Of the two sown species that were most dominant in our plots, only one was affected by seed mix design (*Ec. purpurea*), a forb commonly sown in prairie restorations. This species had higher cover in high‐source diversity fields. Although this increase in cover was not present in fields with high species diversity, we suspect this is caused by this species being sown at a lower rate in this seed mix and would have otherwise had comparable levels of cover. This indicates that outside of the preferential consumption of some seed sources, there may be some species‐specific advantages to existing in populations with higher source diversity. While the mechanism driving this increase in cover requires further study, we hypothesize that increased source diversity of *Ec. purpurea* resulted in greater niche width, reducing intraspecific competition and increasing cover, as has been observed in other studies (e.g., Crutsinger et al., 2008).

### Seed mix design effects were strongest during initial establishment

One of the clearest effects in this study is that seed mix design decisions have their greatest effect during the first year of establishment, with those effects dissipating over time. During the first growing season, the species that arrived in each plot were clearly determined by the seed mix added to the site. By the fifth year, though, non‐sown species established and competitive interactions between those species were likely the primary drivers of community structure. While it may appear that seed mix design decisions do not have long‐term consequences, there are alternatives that should be considered. First, since the effects of seed mix diversity are scale dependent, with clearer patterns emerging at larger scales (Catano et al., 2021). Thus, some of the effects that we observed at the plot scale in the first year may have still been present 5 years later, but only apparent at larger scales. Additionally, sown species were often rare in our plots, especially those sown with high species diversity seed mixes. Had there been a greater abundance of sown species in our experiment, the effects of seed mix design may have been more apparent.

Moreover, since we only measured plant communities at two timepoints, we may have missed patterns related to inter‐annual variation, especially those driven by climate. Previous work in grassland systems has demonstrated that precipitation can have a large effect on annual turnover in plant communities, especially for rare species (Cleland et al., 2013; Groves et al., 2020). Our sites (Michigan, USA) experienced higher than average precipitation during many of the survey years, especially during the planting year (PRISM climate group, 2022; Appendix S1: Figure S3). This wetter climate may have bolstered the cover of non‐sown species (Groves et al., 2020), suppressing the abundance of sown species. Thus, the sown species that were present when we surveyed may not represent the communities we will observe in the future. Given the call to better understand the long‐term dynamics of restoration decisions (Kaul & Wilsey, 2021), our experiment will provide invaluable opportunities to continue to monitor these plant communities into the future.

### Conclusions

Overall, our results exemplify the importance of accounting for site‐specific factors in restoration research. Had we not accounted for edge effects and consumer access in our experiment, our data would suggest that modifying sown species diversity largely had no effect on restored plant communities. Instead, our results indicate that there are some conditions in which seed mix design is likely to influence restoration outcomes. For example, if planting in an area with large amounts of edge or high consumer pressure, increasing the number of species in a seed mix may reduce those effects. However, this will only be true if the seed mix retains high seeding rates of reliably establishing species. Increasing source diversity may have neutral effects in fields with low consumer pressure (and perhaps benefit some species such as *Ec. purpurea*), but detrimental impacts in fields with more consumers. Our study indicates that restoration outcomes may be driven both by the decisions that managers make as well as the environmental conditions the communities establish in and provides potential drivers for variation in restoration outcomes.

## AUTHOR CONTRIBUTIONS

Lars A. Brudvig and Nash E. Turley conceived the ideas. Riley B. Pizza, Lars A. Brudvig, and Nash E. Turley designed the methodology. Nash E. Turley and Riley B. Pizza collected the data. Riley B. Pizza analyzed the data and led the writing of the manuscript. All authors contributed critically to the drafts and gave final approval for publication.

## CONFLICT OF INTEREST STATEMENT

The authors declare no conflicts of interest.

## Supporting information


Appendix S1:

## Data Availability

Data and code (Pizza et al., 2024) are available in Dryad: https://doi.org/10.5061/dryad.0vt4b8h71.
