# Supplementary material for: Relative effects of seed mix design, consumer pressure, and edge proximity on community structure in restored prairies
Source: Ecol Appl. 2025 Jan 19;35(1):e3083. doi: 10.1002/eap.3083 (PMC11744057; doi:10.1002/eap.3083)
Supplement: Supplementary file 1 — Appendix S1: [file EAP-35-e3083-s001.pdf]

Ecological Applications

Appendix S1

Relative effects of seed mix design, consumer pressure, and edge proximity on community structure in restored prairies

Riley B. Pizza, Nash E. Turley, Lars A. Brudvig

Table S1: Species lists and seeding rates (seeds/m^2) for both the low and high species diversity seed mixes. Source information was only available for the focal species where source was manipulated in the experiment. For sites where source diversity was high, each source represents ~1/3 of the total number of seeds sown.

Species	Low species diversity mix seeds/m^2	High species diversity mix seeds/m^2	Local source	Midwest Source	Southern Source
Andropogon gerardii	48.87	22.28	Wexford Co., MI	Kenosha Co., WI	Macon Co. MO
Bouteloua curtipendua	44.45	22.28	IL	Green Co., WI	Livingston Co., MO
Elymus canadensis	44.45	24.43	Newton Co., IN	Waushara Co., WI	IA
Koeleria macrantha	44.45	24.76	Wexford Co., MI	Columbia Co., WI	IA
Schizachyrium scoparium	49.41	22.28	St. Joseph Co., MI	Columbia Co., WI	MO zone 1 ecotype
Chamecrista fasciculata	16.36	2.15	Lucas Co., OH	Grant Co., WI	Cole Co., MO
Coreopsis lanceloata	13.89	3.23	Newton Co.,IN	Wi	Joplin Co., MO
Dalea purpurea	11.41	2.69	Ontario, Canada	Dakota Co., MN	MO
Echinacea purpurea	13.35	3.23	Lucas Co., OH	Madison, IA	Putnam Co., MO
Lespedeza capitata	16.36	2.80	OH	Whiteside Co., IL	Miller Co., MO
Ratibida pinnata	16.36	3.23	OH	Madison, IA	Greene & Hickory Co., MO
Rudbeckia hirta	10.66	2.26	OH	Kenosha Co., WI	Barton Co., MO
Bromus kalmii		15.82			
Carex bicknellii		4.20			
Carex brevior		7.21			
Carex granularis		1.94			
Carex molesta		6.14			
Carex muhlenbergii		1.51			
Carex normalis		3.12			
Carex vulpinoidea		24.76			
Elymus trachycaulus		10.23			
Elymus virginicus		6.24			
Panicum virgatum		13.89			
Sorghastrum nutans		41.55			

Spartina pectinata	4.84
Sporobolus cryptandrus	49.41
Sprobolus heterolepis	7.86
Agastache nepetoides	5.60
Allium cernuum	1.83
Aquilegia canadensis	2.37
Arnoglossum atriplicifolium	1.51
Asclepias tuberosa	1.08
Asclepias verticillata	1.40
Astragalus canadensis	4.20
Baptisia lactea	0.86
Coreopsis tripteris	1.72
Desmanthus illinoensis	0.54
Desmodium canadense	0.86
Desmodium illinoense	0.54
Potentila arguta	14.21
Eryngium yuccifolium	3.66
Gaura biennis	1.29
Helianthus occidentalis	1.72
Heliopsis helianthoides	1.51
Liatris aspera	1.94
Liatris cylindracea	1.72
Lupinus perennis	0.54
Monarda punctata	5.60
Parthenium integrifolium	0.86
Penstemon digitalis	8.07
Penstemon hirsutus	7.75
Pycnanthemum tenuifolium	23.36
Rudbeckia triloba	4.20
Senna hebecarpa	0.65
Silphium integrifolium	0.65
Silphium laciniatum	0.32
Silphium terebinthinaceum	0.22

Solidago rigida	2.58
Solidago speciosa	5.92
Symphyotrichum ericoides	12.38
Symphyotrichum laeve	13.56
Symphyotrichum novae-angliae	4.09
Symphyotrichum oolentangiense	9.90
Tephrosia virginiana	0.65
Thaspium trifoliatum	2.26
Tradescantia ohiensis	0.97
Verbena stricta	3.44
Vernonia fasciculata	3.01
Veronicastrum virginicum	49.41
Zizia aurea	1.40

Table S2. Beta values and standard errors for the response variables used to quantify the effects of seed mix diversity, edge effects, and consumer pressure on restored prairie plant communities. Experimental plots were measured during early establishment (2016) and five years later (2021) to identify persistence. †p < 0.10. *p < 0.05.; **p < 0.01.; ***p < 0.001.

	**Species richness**				**Evenness**				**Community diversity**				**Sown Richness**				**Sown Cover**				**Focal Richness**				**Focal cover**			
	2016		*2021*		*2016*		*2021*		*2016*		*2021*		*2016*		*2021*		*2016*		*2021*		*2016*		*2021*		*2016*		*2021*	
	**β**	**SE**	**β**	**SE**	**β**	**SE**	**β**	**SE**	**β**	**SE**	**β**	**SE**	**β**	**SE**	**β**	**SE**	**β**	**SE**	**β**	**SE**	**β**	**SE**	**β**	**SE**	**β**	**SE**	**β**	**SE**
Exclosure	0.05	0.09	-0.08	0.09	-0.05	0.12	0.01	0.14	0.01	0.10	-0.07	0.11	**0.45** ***	0.13	0.01	0.12	0.08	0.08	0.11	0.09	**0.54** ***	0.12	-0.01	0.09	**0.39** ***	0.11	-0.02	0.11
Center/edge	-0.15	0.17	**-0.26** †	0.15	0.02	0.18	0.09	0.18	-0.06	0.17	-0.10	0.20	**-0.45** **	0.14	-0.18	0.18	-0.16	0.18	-0.24	0.23	**-0.42** **	0.14	-0.21	0.20	**-0.25** †	0.15	-0.18	0.18
Species diversity (SD)	0.07	0.23	-0.36	0.23	-0.10	0.19	-0.04	0.19	-0.01	0.18	-0.26	0.21	0.09	0.23	0.10	0.25	0.37	0.22	0.21	0.23	**0.44†**	0.21	**0.47** *	0.23	**0.62** **	0.21	0.31	0.25
Seed source Diversity (SSD)	0.14	0.26	0.09	0.25	-0.22	0.25	-0.13	0.23	-0.09	0.26	-0.06	0.22	0.09	0.23	0.29	0.25	0.01	0.23	-0.07	0.25	0.16	0.21	0.20	0.24	0.34	0.21	0.37	0.25
Water holding capacity	-0.18	0.12	-0.19	0.12	-0.09	0.11	-0.03	0.11	-0.13	0.10	**-0.24** *	0.12	0.07	0.13	-0.04	0.13	-0.08	0.11	-0.14	0.12	0.05	0.11	-0.07	0.12	-0.05	0.11	-0.04	0.13
Exclosure*SD	-0.05	0.09	0.08	0.09	0.08	0.12	0.20	0.14	0.01	0.10	**0.27** *	0.12	**-0.27** *	0.13	-0.02	0.12	0.05	0.08	0.03	0.10	**-0.33** **	0.12	-0.04	0.09	-0.12	0.11	0.10	0.11
Exclosure*SSD	-0.05	0.09	-0.07	0.09	0.13	0.12	0.22	0.14	0.08	0.10	0.07	0.12	**-0.21** †	0.13	-0.15	0.12	-0.02	0.08	-0.15	0.09	**-0.24** *	0.12	**-0.17** †	0.09	**-0.25** *	0.11	-0.16	0.11
Center/edge* SD	-0.16	0.18	0.17	0.15	**-0.37** *	0.18	-0.11	0.18	**-0.33** *	0.17	-0.01	0.20	0.04	0.14	-0.11	0.19	**-0.38** *	0.18	-0.18	0.23	-0.08	0.14	-0.15	0.20	-0.17	0.15	0.02	0.18
Center/edge *SSD	0.10	0.17	0.20	0.15	0.17	0.18	0.10	0.18	0.20	0.16	0.25	0.20	0.19	0.14	0.11	0.18	0.13	0.18	0.21	0.23	**0.27†**	0.14	0.05	0.20	0.22	0.15	-0.06	0.18
SD* SSD	0.07	0.25	-0.12	0.25	**0.29** †	0.18	-0.22	0.18	0.24	0.17	-0.21	0.21	0.10	0.25	-0.25	0.26	0.26	0.24	-0.11	0.23	0.04	0.23	-0.11	0.24	-0.12	0.22	-0.28	0.27

Table S3. Beta values and standard errors for the cover of 6 common species (found in at least half of our plots) used to quantify the effects of seed mix diversity, edge effects, and consumer pressure on restored prairie plant communities. Experimental plots were measured during early establishment (2016) and five years later (2021) to identify persistence; some species that were common in 2016 were not common in 2021, and visa-versa. †p < 0.10. *p < 0.05.; **p < 0.01.; ***p < 0.001.

	Elymus Repens				***Solidago Canadensis***				***Daucus Carota***		***Plantago Lanceolata***		***Andropogon gerardii***		***Poa pratense***		***Echinacea purpurea***	
	2016		*2021*		*2016*		*2021*		*2016*		*2016*		*2021*		*2021*		*2021*	
	**β**	**SE**	**β**	**SE**	**β**	**SE**	**β**	**SE**	**β**	**SE**	**β**	**SE**	**β**	**SE**	**β**	**SE**	**β**	**SE**
Exclosure	**0.87***	0.32	0.15	0.21	0.34	0.30	-0.52	0.38	-0.27	0.41	-0.45	0.30	0.60	0.37	0.52	0.64	0.54	0.93
Center/edge	0.28	0.73	1.13	0.85	-0.89	0.55	-0.13	0.80	0.05	0.74	-0.04	0.53	-1.44	1.55	-0.27	0.67	0.84	1.36
Species diversity (SD)	0.86	0.78	-0.61	0.95	0.45	0.55	0.28	0.86	0.47	0.77	-0.62	1.21	1.16	2.12	-0.52	0.81	**3.70****	1.42
Seed source Diversity (SSD)	**2.25***	1.08	0.28	1.37	-0.32	1.11	0.71	1.20	1.63	1.02	-1.19	1.41	-1.10	2.13	0.46	0.88	**2.93***	1.38
Water holding capacity	0.37	0.25	0.01	0.25	-0.21	0.21	-0.21	0.24	0.02	0.22	-0.06	0.28	-0.18	0.37	-0.22	0.22	**-1.03 ****	0.37
Exclosure*SD	-0.25	0.34	**-0.61***	0.29	-0.30	0.33	0.22	0.42	0.01	0.42	0.35	0.38	-0.39	0.46	-0.15	0.68	0.44	0.91
Exclosure*SSD	**-0.84***	0.35	0.21	0.28	**-0.55†**	0.32	0.15	0.42	0.38	0.42	0.19	0.34	-0.30	0.44	-0.60	0.68	**-1.71***	0.76
Center/edge* SD	-0.41	0.79	-0.51	0.98	-0.22	0.66	-0.71	0.90	-0.09	0.79	**1.38***	0.65	-1.94	1.79	0.66	0.76	-1.77	1.38
Center/edge *SSD	-0.98	0.81	**-1.73†**	1.01	0.74	0.64	-0.29	0.91	-0.23	0.79	-0.58	0.66	0.39	1.78	0.15	0.74	-1.88	1.31
SD* SSD	-0.16	0.82	**2.53***	1.03	-0.07	0.65	-0.11	1.01	-1.07	0.84	1.87	1.65	1.00	2.84	0.21	0.76	**-2.92***	1.38

Table S4. Partial regression coefficients and F statistics for two PERMANOVA analyses used to quantify the effects of seed mix diversity, edge effects, and consumer pressure on the community composition of restored prairie plant communities. Model 1 was used to understand the effects of seed mix diversity on community composition, and model 2 was used to understand the impact of edge effects and consumer pressure, as well as their interaction with species diversity of a seed mix. Plots were measured during early establishment (2016) and five years later (2021) to identify persistence; *p < 0.05.; **p < 0.01.; ***p < 0.001.

	**Model 1**				**Model 2**			
	2016		***2021***		***2016***		***2021***	
	R^2	*F*	R^2	*F*	R^2	*F*	R^2	*F*
Seed source diversity (SSD)	0.04	**8.49*** **	0.02	**4.01** **	---	---	---	---
Species diversity (SD	0.02	**4.42*** **	0.02	**3.00*** **	0.02	**4.54*** **	0.02	**3.06*** **
SSD*SD	0.02	**3.65*** **	0.02	**3.53*** **	---	---	---	---
Water holding capacity (%)	0.02	**5.12*** **	0.04	**7.18*** **	0.02	**5.13** **	0.04	**7.66*** **
Site/half	0.19	**3.98*** **	0.17	**3.21*** **	0.21	**4.01*** **	0.19	**3.27*** **
Exclosure	---	---	---	---	0.01	0.19	0.01	1.27
Center/edge	---	---	---	---	0.02	**3.74*** **	0.01	**2.02* **
Exclosure*SD	---	---	---	---	0.01	0.47	0.01	0.64
Center/edge* SD	---	---	---	---	0.01	**2.11* **	0.01	**1.76* **

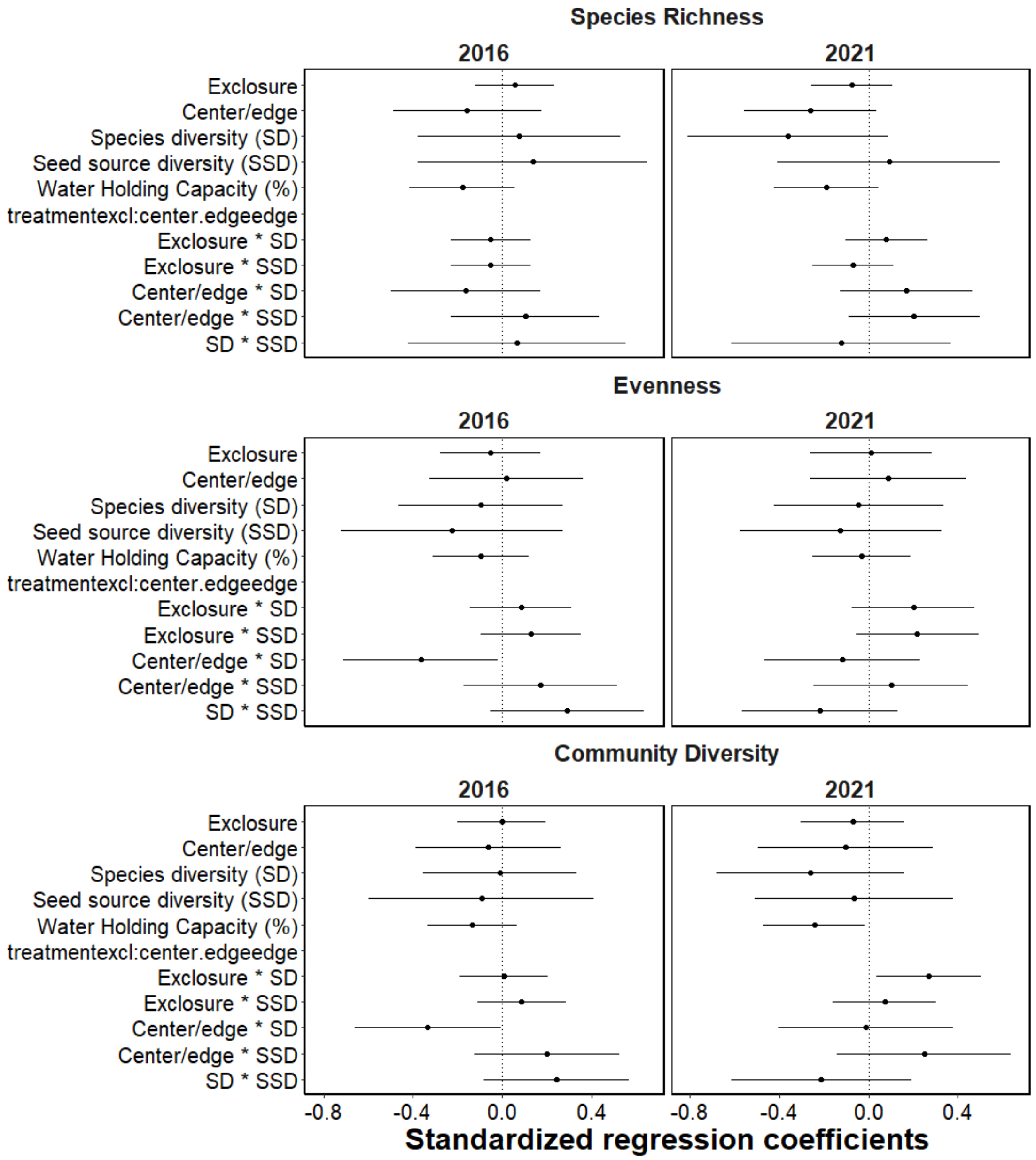

Figure S1: Standardized regression coefficients and 95% confidence intervals for three response variables measured in 2016 and 2021 to quantify the effects of seed mix design, edge effects, consumer pressure, and their interactions, on community composition of tallgrass prairie sites undergoing restoration.

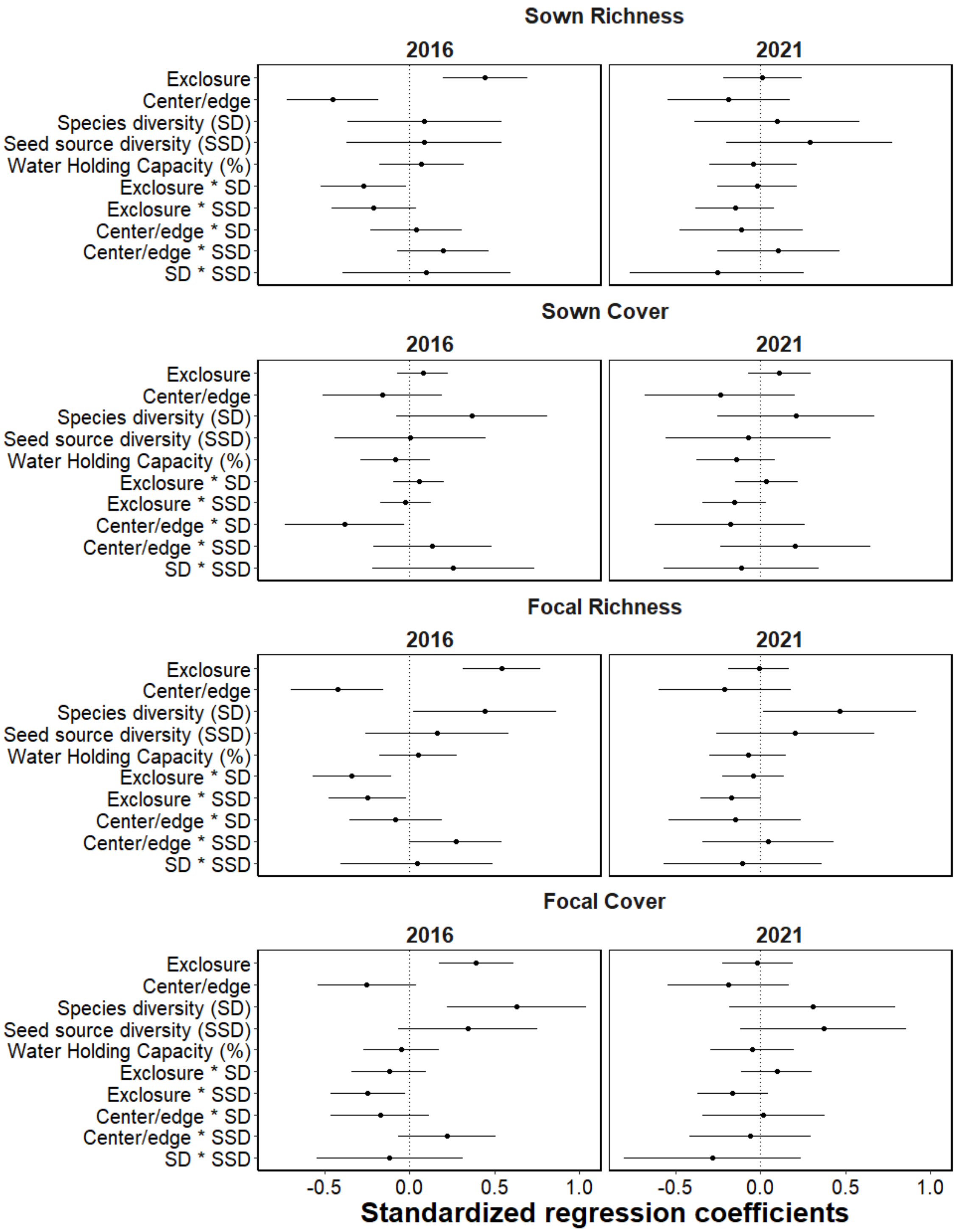

Figure S2: Standardized regression coefficients and 95% confidence intervals for four sown species response variables measured in 2016 and 2021 to quantify the effects of seed mix design, edge effects, consumer pressure, and their interactions on the presence and abundance of species

sown into tallgrass prairie sites undergoing restoration “Sown” refers to any species that were included in either the low or high species diversity seed mix, and “Focal” refers to the 12 species included in both seed mixes where seed source diversity was manipulated.

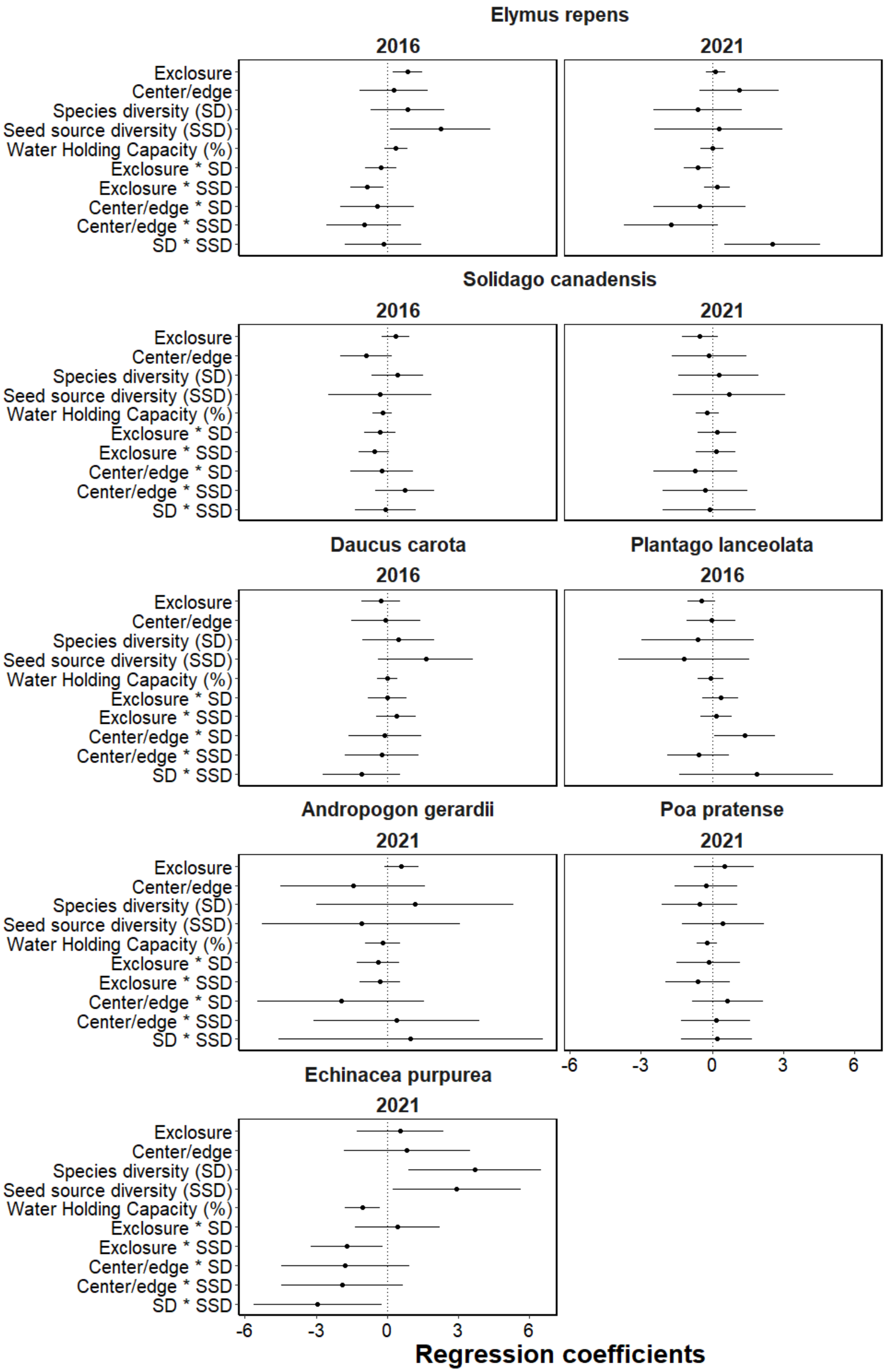
Elymus repens
Solidago canadensis
Daucus carota
Plantago lanceolata
Andropogon gerardii
Poa pratense
Echinacea purpurea
2016
2021
Exclosure
Center/edge
Species diversity (SD)
Seed source diversity (SSD)
Water Holding Capacity (%)
Exclosure * SD
Exclosure * SSD
Center/edge * SD
Center/edge * SSD
SD * SSD
-6
-3
0
3
6
Regression coefficients

Figure S3: Regression coefficients and 95% confidence intervals for the % cover of 6 common species (found in at least 45 of our plots) measured in 2016 and 2021 to quantify the effects of seed mix design, edge effects, consumer pressure, and their interactions on the presence and abundance of common species into tallgrass prairie sites undergoing restoration. Some species that were common in 2016 were not common in 2021, and visa-versa. Due to limitations of this modeling package, coefficients are not standardized.

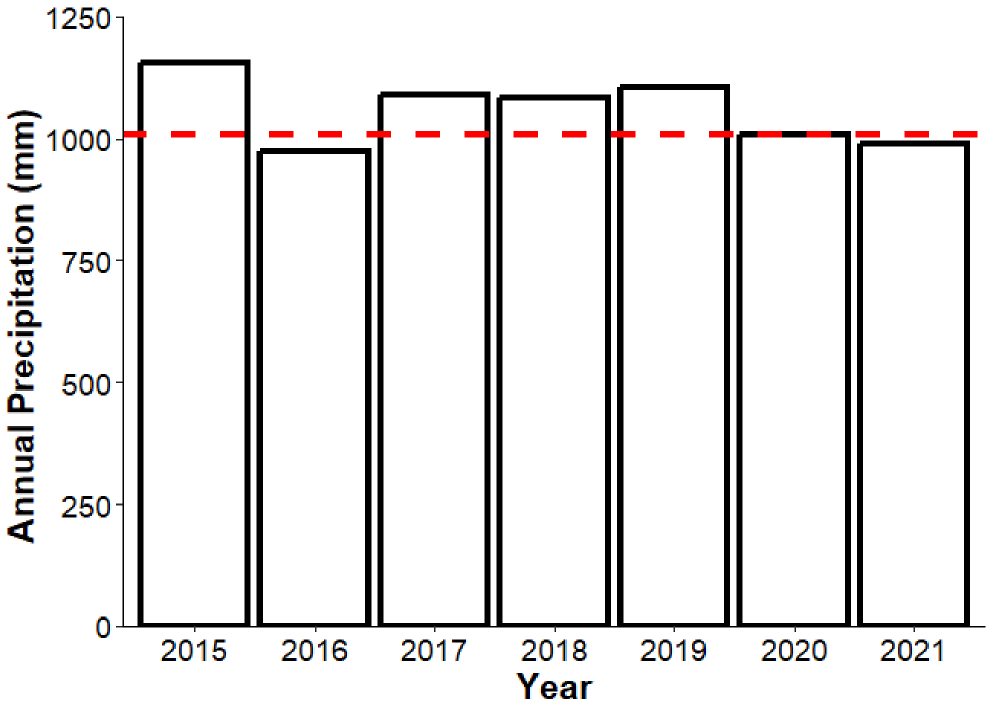

Figure S4: Annual precipitation values for the years our experiment ran: 2015 was the year of the first survey and 2021 was the year of the second. The red dashed line represents the 30-year average precipitation values from the PRISM climate database. Climate data were obtained from a weather station at the NSF Long-term Ecological Research Program at the Kellogg Biological Station and by Michigan State University AgBioResearch.
